# Measuring Life Events and Their Association With Clinical Disorder: A Protocol for Development of an Online Approach

**DOI:** 10.2196/resprot.4085

**Published:** 2015-07-14

**Authors:** Ruth Spence, Amanda Bunn, Stephen Nunn, Georgina M Hosang, Lisa Kagan, Helen L Fisher, Matthew Taylor, Antonia Bifulco

**Affiliations:** ^1^Centre for Abuse and Trauma StudiesMiddlesex UniversityLondonUnited Kingdom; ^2^Department of PsychologyMiddlesex UniversityLondonUnited Kingdom; ^3^Department of PsychologyGoldsmiths University of LondonLondonUnited Kingdom; ^4^Institute of PsychiatryPsychology & NeuroscienceKing's College LondonLondonUnited Kingdom

**Keywords:** disorder, interview, life events, online systems, stress

## Abstract

**Background:**

Severe life events are acknowledged as important etiological factors in the development of clinical disorders, including major depression. Interview methods capable of assessing context and meaning of events have demonstrated superior validity compared with checklist questionnaire methods and arguments for interview approaches have resurfaced because choosing the appropriate assessment tool provides clarity of information about gene-environment interactions in depression. Such approaches also have greater potential for understanding and treating clinical cases or for use in interventions.

**Objective:**

(1) To argue that life events need sophisticated measurement not satisfactorily captured in checklist approaches. (2) To review life-events measures and key findings related to disorder, exemplifying depression. (3) To describe an ongoing study with a new online measure and to assess its psychometric properties and the association of life events in relation to disorder and educational outcomes.

**Methods:**

The Computerised Life Events Assessment Record (CLEAR) is under development as a tool for online assessment of adult life events. Based on the Life Events and Difficulties Schedule interview, CLEAR seeks to assess life events to self and close others, link these to other events and difficulties, and utilize calendar-based timing, to improve upon checklist approaches.

**Results:**

The CLEAR study is in the preliminary stages and its results are expected to be made available by the end of 2015.

**Conclusions:**

There is currently no sophisticated technological application of social risk factor assessment, such as life events and difficulties. CLEAR is designed to gather reliable and valid life-event data while combating the limitations of interviews (eg, time consuming and costly) and life-event checklists (eg, inability to accurately measure severity and independence of life events). The advantages of using such innovative methodology for research, clinical practice, and interventions are discussed.

## Introduction

### Overview

Links between life events and clinical disorders have a long history, given the fact that stressful life events are an important predictor of the onset and course of various disorders across the life span, including depression, eating disorders, and psychosis [1-5]. In addition, long-term stressors (difficulties) play an important role in the onset and maintenance of disorder, notably depression, but these are often overlooked [6,7].

Empirical investigation of life events and disorder started with checklist self-report approaches in the 1960s [8], but the field was invigorated by the introduction of investigator-based interviews from the 1970s onward by Brown and Harris [9], with the Life Events and Difficulties Schedule (LEDS) Interview [9], and by Paykel [10] and Dohrenwend et al [11]. This paper is mainly concerned with the LEDS approach, although some points will equally apply to other interview measures as well. The LEDS focused on contextually assessed life events: first to incorporate the likely meaning of the event for an individual rather than using a generic scoring system, and second to avoid bias in reporting due to depressed mood and making sense of an illness episode retrospectively [12]. Although such approaches added to the complexity while improving the validity of life-event measurement, they invoked high costs in researcher and participant time as well as in researcher training. This has led to the use of checklist approaches in recent years [13], especially in the search for gene-environment interactions (GxE) in depression, because these studies require large sample sizes.

This paper outlines the ineffective measurement of life events in many contemporary research studies. It also presents a new online computerized approach—Computerised Life Events Assessment Record (CLEAR)—designed to optimize interview advantages while incurring low cost and being time effective. The ongoing development and future testing of CLEAR will be outlined with a focus on clinical health. It is expected that this new online method will offer an enhanced but readily available life-event measure with important implications for studying disorders.

The development of CLEAR has implications for genetic studies of depression as well as for more effective clinical application. For instance, some individuals are more likely to experience severe life events, because of psychosocial vulnerability (eg, difficulty in relationships resulting in more relationship events) [14]; likewise, based on similarities observed in twins [15,16], it appears that some individuals select themselves into high-risk situations due to genetic or familial factors [17,18]. Here the measurement of life events has proved critical, with genetic studies producing inconsistent findings for GxE in depression [19]. Thus, while several large studies have found a significant relationship between GxE for the serotonin transporter polymorphism (*5-HTTLPR* genotype) and life events in depression [20,21], others have failed to do so [22,23]. Uher and McGuffin [24] pointed out that the failures to replicate GxE results are more common in studies using checklist life-event questionnaires rather than interviews. Certainly, studies that have elicited stressful life events using more involved methodologies (eg, life-history calendar or interview) have tended to find significant interactions between life events and the *5-HTT* gene [25,26]. Thus, the current research demand for more sophisticated measures of life events lies in the genetic field, which would also aid any study requiring large sample sizes, clinical assessment, and treatment interventions.

### Life-Event Interviews and Questionnaires

Among the different in-person semistructured interviews, probably the most widely used is the LEDS [9,27]. This approach encourages narrative accounts of events that can elicit the full social context, their timing, and sequence in relation to disorder onset. LEDS encapsulates a large range of events to the self and close others. It deals with the likely meaning of events by collecting contextually relevant information (both biographical and current circumstances) and rates according to precedent examples, stripped of subjective response.

This interview is considered the “gold standard” for measuring life stress and is superior to checklist approaches. The disadvantage comes from the time and labor involved [28] in analyzing the numerous constructs rated and the algorithms required (eg, for “severe event” definitions). For example, the LEDS interview takes 1-2 hours, but up to 16 hours to complete with full ratings and checking [29]. This incurs high costs and places a burden on the interviewer, making it an unattractive alternative to checklists for most studies [30]. Thus, there is a need for an approach that has the reliability and validity benefits of such comprehensive face-to-face interviews while being more economical.

### Key Features of Life Events and Measurement Issues

#### Events and Change

The early investigation of life events by checklist (eg, Holmes and Rahe questionnaire [8]) characterized “a life change unit” as the main element with generic scoring of stressfulness routinely applied to events. Thus, “death of a spouse” was given the highest stress weighting (100), and minor violations of the law given the lowest (11). This approach makes 2 assumptions, which we challenge: first that life events require routine practical change and second that the stressfulness valence can be decided generically. In terms of change, we agree that the more extensive and permanent the negative life change, the more likely it is to invoke a stress response. Thus, permanent negative changes (eg, death of a spouse) get the highest ratings in this self-report, with routine and conditional change being rated the lowest (eg, begin or end school or college). However, this scheme has a pedestrian view of change as an observable shift in routine. In real life, however, degree of change is often not known at the commencement of an event (eg, partner leaves home after a row), or the change is definite but has not yet occurred (eg, forecast of redundancy), or news of the event occurs after the change has happened (eg, death of a relative abroad). Some of the most damaging events present no immediate practical change (eg, betrayal in a close relationship) but require substantial cognitive reappraisal. It is also important to ask, “Change to whom?.” Events to close others, particularly those experienced jointly with the self can also have highly stressful impacts (eg, partner’s severe illness requiring the respondent’s caring responsibility). These are not usually included in self-report approaches. The LEDS covers events in 12 different domains, with up to 10 subdivisions in each, as well as routinely covering events to self and to a range of predetermined close others [27]. Thus, the array of events included is vastly higher and arguably captures a more realistic range of stressful experiences.

#### Context and Severity

The other aspect involves the estimated severity of the event in terms of a likely stressful and negative emotional response in most people. In checklist approaches, this is generically ascribed. Yet, apart from the worst ones (eg, death of a spouse), almost all are dependent on context for their likely severity. For example, marriage and pregnancy are not inherently stressful unless the context is negative (eg, unplanned pregnancy, unstable partnership, health risks, or financial and housing difficulties), where a much higher stress score is allocated. A more recent checklist identified those events most often scored as severe life events in interview measures [13] and included events to close others, but the scoring of events is still generic rather than context dependent. Yet a study by Dohrenwend and colleagues [31] found that the lack of context contained within questionnaire measurement hid response heterogeneity. There was high variation in what respondents classified under each event and they often elicited trivial events [29]. Therefore, questionnaire categories can mask important differences in responding. Life-events checklists ultimately provide a total score based on the number of items endorsed, sometimes with a weighting applied. They do not assess the severity of each event experienced, with a view to one event being able to predict disorder. In interview measures such as the LEDS, context is determined by careful questioning about circumstances leading to and surrounding the event, with salient aspects included into the event context for judging severity. All of these contextual factors are objectively classified, not dependent on the emotional response of the individual.

#### Meaning of Events: Loss, Danger, and Humiliation

Interview measures have found that the likely meaning of an experience plays a central etiological role in the development of depression, with life events tied to changes involving loss (of relationship, role, cherished idea, or sense of self), danger (threat of a future loss, conflicts in core social roles, threats to plans you have made), or punishing environments (entrapment, humiliation) being the most predictive of disorder [1,7]. Equally, an individual’s plans and concerns need to be considered; an event may derail long-term plans or undermine a role involving behavioral commitment (eg, caring mother, diligent student, dedicated worker). One prospective LEDS study found that a “severe event” in a life domain of previously determined high commitment more than doubled the risk of a depressive episode when compared with others in areas of lower commitment [6]. A further study showed that specific attributes make events more predictive of disorder: humiliation and entrapment [7]. “Humiliation” is an event involving a put down, devaluation, or rejection, and “entrapment” confirms imprisonment in an ongoing, highly punishing situation involving a chronic stressor or difficulty [7]. Entrapment events additionally predict comorbid depression and anxiety [7,32], as well as relapse of depression [33] and operate cross-culturally [34]. Therefore, a full determination of an event’s capacity to provoke a depression requires careful exploration and scoring of the salient experience including recent plans and behavioral commitment [7,35]. Questionnaire approaches tend to lack this depth and clarity, and therefore, underestimate the presence of stressors by overly summarizing the range of events possible without attention to such attributes.

### Timing and Chronicity of Stressors

The timing of events is critical to determining their etiological role in depression onset. Events that occur after onset can only have a maintenance role at best. Therefore, precise timing of events is required. In addition, other important stressors are chronic, with severity levels that can vary over time. These are termed “difficulties” and comprise problematic situations, which last 4 weeks or more, and can go on for years. These can occur in as many domains as events, and can be antecedent or consequent to the event. An important analysis of such linkages showed that an event preceded by a severe difficulty (hence “matched”) for at least 6 months and in the same domain greatly increased the risk of depression onset [6]. In this case, the potential for entrapment or an erosion of hope can add to the burden of the ongoing problem (eg, a partner’s demand for a divorce in the context of a conflictual marriage; or a failed attempt at rehousing in the ongoing problem of serious overcrowding). Using these criteria, women with a severe event “matching” a difficulty had a threefold greater chance of developing depression [6]. Questionnaire measurement cannot reflect such links and is imprecise regarding the timing of the event in relation to onset of disorder. Severe events of etiological importance occur within 6 months of onset and often within half of that time [35]. In addition, the effects of life events gradually decay over time, with the strongest effect in the month immediately following the life event with some variation by event type [2,36]. Without knowing the timing of events, any precision is lost, which restricts the causal attribution of life events to disorder [37] and the investigation of specific stressors for different disorder outcomes [38].

### Independence From Individual’s Own Actions

Life-event interviews also categorize “independence” of the event. This is the extent to which the event is likely to be separate from the actions, planning, or control of the individual, that is, it occurs externally to the individual. Independence allows researchers and clinicians to estimate whether the event is a cause or consequence of disorder. For example, losing a job because the employer has gone bankrupt would be judged totally independent outside of personal control; personal health events are “nearly totally” independent, interactions with close others only “possibly independent,” and intentional acts as “nonindependent” [9]. Events that are a part of the depression itself or its treatment (suicide attempt or psychiatric hospital admission) are rated as “least independent” and termed “illness related.” Genetically sensitive twin studies of depression and life events have described genetic influences for nonindependent events, but not for independent events [39]. Both relate to depression.

Given this context, the inadequacy of checklist life-event questionnaires for etiological study of depression is apparent. Although quick and easy to administer, requiring few resources, they are subject to serious methodological limitations compromising the quality of the data gathered.

### Need for a New Approach

Digital health interventions are increasingly seen as a way to assess, treat, and prevent psychological disorder and deliver mental health provision. Such Web-based assessments and services have the ability to overcome geographical barriers, lower delivery costs, and reduce workforce demands [40]; in addition, the systems are convenient, assessments can be answered anonymously, and personalized feedback can be provided [41]. They can also provide avenues of research into processes related to mental health and well-being [42]. While digital health is a rapidly expanding area of research and practice, there is no sophisticated technological application of social risk factor assessments (such as life events and difficulties) that can benefit from many of the same advantages. There are, however, online measures with precoded algorithmic scoring used successfully within research for psychiatric diagnoses in children and adolescents (eg, Development and Well-Being Assessment, [43]) and adults (eg, OPCRIT, [44]) and for highlighting individuals at risk of physical illness such as Parkinson’s disease (eg, PREDICT-PD, [45]). Such tools have also aided assessment with vulnerable children [46]. Thus, it seems likely that complex social risk factors could be measured in the same way.

The current project in progress aims to address the need for improved and accessible life stress measurement by developing an online data capture tool (CLEAR) and testing its psychometric properties and its association with disorder and educational outcomes. Currently, the project is in its early stages and CLEAR is still under development. In the following sections, we outline the basic architecture of the CLEAR system and the study to test it once complete.

## Methods

### Participants

CLEAR is a new complex measurement tool, and therefore, its feasibility and usability will be assessed by life-event expert and nonexpert volunteers (n=20) across a range of ages. These groups will act as a panel to test out CLEAR before it is finalized. Panelists will rate either their own experience, or case study examples from archived interview data, to determine both user friendliness and whether the full context of the event can be adequately captured. Their feedback will inform improvements to the system.

The project will utilize 3 different samples to develop and test CLEAR. A midlife sample (average age 52) will be recruited from the Depression Case Control (DeCC) study, involving a pool of 2299 respondents from London, Cardiff, and Birmingham, originally studied for gene-stress interaction and depression [47]. Those with prior recurrent depression will be assessed by clinical interviews (n=125) and unaffected controls (n=125) will be reapproached for the study. Half of the depressed cases will be selected based on having previously reported a lifetime common illness (asthma, hypertension, osteoarthritis, and thyroid problems), as will 25% of the controls (31/125) consistent with original prevalence rates. Furthermore, 125 1st-year undergraduates (average age 19) will be included for studying educational outcomes. This will test whether CLEAR can capture life events during different life stages. In addition, it will add to the limited evidence base regarding whether life events are related to student performance [48,49]. This is an important area to understand as students show higher rates of depression [50], and younger adults, in general, experience a higher rate of life events [51]. Developing a greater understanding of their experience can help with providing improved support at this critical life stage, which may have a lasting impact on future opportunities.

### Procedure and Analysis

Participants will be approached by letter or email, which will explain the study and enclose an information sheet and consent form. Those who are interested in the study will be sent the CLEAR URL and log-on details, which will allow them to access and complete CLEAR from any Internet-enabled computer or tablet. The validity of CLEAR will be assessed by interviewing 30 participants from each of the samples (10 undergraduates, 10 recurrent depression cases, and 10 unaffected controls) using the in-person LEDS interview and CLEAR in counterbalanced order. The time taken for each participant to complete CLEAR will depend to some extent on how many life events have occurred over the 12-month period. However, the average in-person LEDS takes approximately an hour to complete, and therefore, it is assumed this will be the average time taken to finish CLEAR.

The data generated will be rated blind/reviewed by separate researchers and compared using Cohen kappa and intraclass correlation coefficients (ICCs) for level of agreement between the 2 methods. The total number of life events captured, the domain category, severity rating, and the timing of events will be compared to give an indication of how well CLEAR mimics the in-person method for full reporting and recall. Any further modifications will be made to CLEAR if required.

Test-retest reliability of CLEAR will be undertaken using an additional 20 undergraduates, 20 depression cases, and 20 controls from each of the samples, measured an optimal 3-4 weeks apart for stability in reporting (using Cohen kappa and ICC). CLEAR will be tested on the remaining participants (n=285) and the rates and types of life events and difficulties reported in the 3 samples will be compared and analyzed in relation to sex, social class, and age using chi-square statistics. The association between life events and past/recent depressive disorder and physical illness (DeCC sample), and academic performance (undergraduate sample) will be tested using logistic regression to look at the contribution of life events and indicators of social disadvantage to health and educational outcomes. Once both reliability and validity of CLEAR have been determined, the program will be available for more general use.

Security is a key concern of CLEAR; the CLEAR servers are built from CentOS Linux 5.4, which is a secure variant of Linux, has no services or ports installed, and includes only what is strictly necessary for CLEAR. In addition, a firewall is installed to further restrict access to the server. All data are entered into CLEAR under a unique log-on, and therefore, no names or contact details are entered on to the CLEAR system. The data are stored on a secure MySQL database that is updated whenever a participant enters information through the CLEAR interface. The log-ons will be stored in a password-protected file with the participant’s study ID numbers. A separate password-protected file will contain the ID numbers and any identifying respondent information (eg, contact details). Therefore, for this study there is the ability to recontact participants if needed.

## Results

The CLEAR study is in the preliminary stages and its results are expected to be made available by the end of 2015.

## Discussion

### CLEAR Instrument

Respondents complete CLEAR by providing demographic information; information about close others; and life events and difficulties over the past year in 12 domains (education, work, reproduction, housing, money, crime, health, romantic, other relationships, children, death, and miscellaneous). The assessment also includes a fixed battery of measures, a depression questionnaire (General Health Questionnaire, GHQ, [52]), and an interpersonal vulnerability questionnaire (Vulnerable Attachment Style Questionnaire, VASQ [53]). However, for projects tailored to other research questions, the integrated calendar system can be used to record events over a greater observation period, and paper or electronic questionnaires can be used in addition to CLEAR. The information is provided through a mixture of checklists for closed answers, text boxes for open-ended answers, and logic-driven checklist menus. CLEAR also contains a feedback system that allows for a personalized calendar, menus, and references to specified close others.

The logic-driven menus guide the respondent based on their prior answers. For example, if a respondent chooses the “education” category, this presents them with a menu of options (eg, selection interview, examination results) and depending on response, a following different set of options is provided (eg, application rejected/accepted versus passed/failed exam). Once respondents have completed these, they describe and score various aspects of the event including the event context, independence, and their feelings about the event. For each event, respondents are asked if this relates to any other event or difficulty and a menu (which continually updates) is presented with all previously entered events and difficulties. Thus, they are also able to link events and difficulties. This creates a dynamic feedback system in which more links between events and difficulties can be added as CLEAR is completed. Throughout this process, there are detailed instructions (including video) and domain-specific examples to inform the respondent. Important aspects, such as level of threat/unpleasantness, are given labels benchmarking the target level to encourage appropriate ratings.

CLEAR is scored using a precoded algorithm to produce a rating of “severe” life event as well as “D-matching” events and other indices. For analysis of the in-person LEDS interview, a derived variable of “severe life event” is one, which is rated (1) “marked” or “moderate” on long-term contextual threat/unpleasantness (ie, objective assessment, present at 10 days after the start); (2) “self” or “joint” focused; and (3) is not “illness related” (ie, part of the disorder investigated such as treatment/hospitalization or symptom related such as suicide attempt). The same algorithm for combining these 3 scales in producing a binary severe life-event variable will be precoded in the CLEAR online version from the data entered and made available for the report produced, or downloading to SPSS (SPSS Inc., Chicago, IL, USA) for further analysis. For “matching difficulty events,” a stated link to rated difficulties of “very marked,” “marked,” or “high moderate” severity is required, in the same domain (eg, work or marital), and of 6 months duration prior to the event. This will similarly be precoded consistent with the regular interview analysis of data.

Information can be pooled from various sources to assign the likely negative meaning of the event for the respondent based on demographics in combination with objective ratings of the event circumstances. The logic-driven menus provide detail about the basic event type and circumstances that may apply (eg, for moving house a submenu is provided where an individual can choose an option “forced to move” and from the following menu tick options that may apply such as “large cost of moving” and “neighborhood less desirable”), and the self-report data provide demographic information including current circumstances (eg, employment status, number of dependents) and historical data (eg, education and employment history). The system also requires self-assessed threat/unpleasantness ratings of events and difficulties. Together, these will be used to produce an overall objective severity rating. In addition, the written descriptions provide further surrounding detail that can be reviewed by researchers to check for reliability. Furthermore, using both the open-ended text-box answers and scores can help researcher review each case in depth, which also allows for quality control checking and enabling extended qualitative analysis if needed, or in a minority of cases recontacting respondents.

The logic-driven menus guide individuals toward the type of events likely to be stressful, from more general to more specific event types. There is evidence to suggest that inclusion of detailed instructions of different event types in each category gives better test-retest reliability with less “fall off” of event reporting over time, and greater agreement between respondent and co-informant [54,55]. Therefore, adopting this approach may help maximize reliability and prevent recall fall off, which will be assessed through a comparison of CLEAR and the in-person LEDS interviews.

Each stem menu of events leads down a path until the options are no longer relevant. However, at each stage, the respondent is given the option of rating “something else/other.” In this way, stressful events that do not fit into proscribed categories or criteria can also be included. This ensures that the specification of events does not make the definition of events too narrow [56].

Difficulties with recall can be a problem for both interview and checklist methodology, even over a 12-month period [24]. Comparisons of longitudinal and cross-sectional studies demonstrate that more events are reported longitudinally than retrospectively [57]. However, using Web-based systems to conduct prospective longitudinal assessments may be a lower resource-intensive method of obtaining detailed descriptions of psychopathology processes over time [58]. In addition, when CLEAR is used retrospectively, recall may be aided as respondents can edit their responses and can complete it over a few sittings and see their own calendar of events before finalizing sequences. Studies have found that respondents who initially fail to report serious events, when given more time to think after initial prompting trigger greater recall or appraisal of the event [59].

Recall is also helped through a personalized calendar that is updated as life events and anchoring anniversaries or social occasions (eg, holidays, birthdays) are added to the system. The timing of important psychopathology-related timings (eg, peak depression) can be added to the calendar. Events are often linked to other events in autobiographical memory; therefore, the use of calendars can lead to better quality (ie, more complete and accurate) retrospective reports of events, even after several years [58,59]. When used in conjunction with self-report methods it improves completeness of the data and dating accuracy [60]. In addition, the use of multiple, self-generated, and personal landmarks further enhances memory [61].

The CLEAR system aims to be as personalized as possible. In general, simple approaches such as addressing individuals by their name can sufficiently personalize a message to heighten attention to the information provided [62]. CLEAR will use the data input, to reflect information back to respondents in a meaningful way. This will include personalization of menu options, such as forenames of close others used to populate the answer options to particular questions (eg, *who was involved in the event*?) or only being presented with certain questions (eg, *what is your partner’s job?*) if they have answered yes to a previous question (eg, *do you have a partner?*). In addition, normative feedback will be presented to summarize and personalize risk and resilience factors based on the information collected. For example, respondents will be given a pre-prepared brief report, which is tailored to their scores on the GHQ and VASQ, as well as a simple calendar of their events when completed. It is hoped that this will increase motivation and enhance the effectiveness of the system at conveying information and improving respondent’s appropriate response.

Lastly, the online system can be completed in private. Compared with interviews, self-administered measures can elicit more events that may be sensitive, embarrassing, or have the potential to bring about negative consequences [30,63]. One study investigating the impact of social anxiety on well-being found that an online survey was able to obtain in-depth qualitative information about delicate or stigmatizing difficulties [64], and adds to a growing literature suggesting that anonymity of the Internet facilitates open discussion of problems, which may be hard to talk about face-to-face [41].

The CLEAR system will also be programmed to provide basic reports for clinicians/health professionals on individuals in health settings with appropriate permissions. Health professionals can be provided with unique log-ons to CLEAR to access the data-generated reports from the database. The reports will provide a summary of each life event (severity score, date, classification from the menus, and written context from the respondent), a calendar denoting sequence and timing, and the scores from the GHQ and VASQ with appropriate description of resulting classification. The data from CLEAR can also be downloaded into SPSS files or specific data can be downloaded based on applied filters (eg, all events in the housing category). The data are a mixture of quantitative variables (eg, event category, threat, age, relationship to person close to them) and qualitative variables (eg, event written description and emotional reaction). The provision of such automated reports, once tested for their informative and useable characteristics, will be a major benefit of the measure to ongoing practice.

### Advantages for Clinicians and Researchers

The first observations concerning life events occurred in the early 20th century in the clinical field when understanding the experience of depressed patients [65], with Meyer the first to create life charts to document events linked to disorder [66]. Thus, clinical approaches as a basis for treatment were an original driver for investigating life events and depression, and a need still exists in modern approaches such as cognitive behavioral therapy [67] where understanding individual appraisal and response to events is critical to effect cognitive and behavioral change. Having easy access to sophisticated measures of life events are therefore of potential help to clinicians and could be used in combination with tailored digital health interventions; for example, cognitive behavioral therapy packages formulated to be used in response to severe life events occurring within an individual’s life.

Severe life events are relatively common, but only a minority of individuals exposed develop depression. Therefore, the role of personal vulnerability is important. Studies including both low self-esteem and negative interpersonal relationships (ie, conflict with partner or child or lack of close support) as vulnerability indices showed interaction effects with stressful life events in the development of depression [68,69]. Certainly, women selected for these vulnerabilities in a prospective study showed 50% risk of new clinical depression onset [68,69]. While the focus of CLEAR is on the provoking agents for depression, additional questionnaires of vulnerability can be included to generate a fuller picture of the individual, with a future prospect of further developing these online.

### Conclusions

This paper argues that life events are complex phenomena not currently served by the most commonly used measurement approach, that is, checklists. This is potentially damaging research investigating the etiology of depression: problematic measurement must surely lead to problematic results. It is hoped that CLEAR’s technological advances will produce a useful compromise between life-event checklists and interview approaches, overcoming some of the limitations of questionnaires while reducing the burden inherent in face-to-face interviews. CLEAR should have the capacity to capture life-event details and context, different attributes of the event, timing of the event, and linkages between events and difficulties. Although it will not provide exhaustive coverage of all possible events, it is presumed that the majority of events will be captured and that most respondents will be able to rate the bulk of their events accurately given the guidance provided through the menus, examples, and appropriate benchmarking. Thus, this new method of measuring life events may be able to gather high-quality data, hopefully with reliability and validity comparable to the gold-standard interview approach, overcoming the problems inherent in relying on checklist approaches in etiological research.

It is also hoped that the CLEAR approach to assessing life events and difficulties will aid those in clinical practice. The provision of recent life charts of events labeled in terms of their likely stressful nature and with attributes relating to loss, danger, humiliation, and entrapment will allow clinicians to consider the level of stressor experienced in seeking to estimate patient appraisal and coping capacity.

## Abbreviations

CLEAR: Computerised Life Events Assessment Record
DeCC: Depression Case Control
GHQ: General Health Questionnaire
GxE: gene-environment interactions
ICC: intraclass correlation coefficients
LEDS: Life Events and Difficulties Schedule
VASQ: Vulnerable Attachment Style Questionnaire
